# Chebulinic acid is a safe and effective antiangiogenic agent in collagen-induced arthritis in mice

**DOI:** 10.1186/s13075-020-02370-1

**Published:** 2020-11-23

**Authors:** Kai Lu, O. Hans Iwenofu, Rita Mitra, Xiaokui Mo, Partha Sarathi Dasgupta, Sujit Basu

**Affiliations:** 1 Department of Pathology, Ohio State University, Hamilton Hall (H166), 1645 Neil Avenue, Columbus, OH 43210 USA; 2 KPC Medical College, Kolkata, 700032 India; 3 Center for Biostatistics, Department of Biomedical Informatics, Ohio State University, Columbus, OH 43210 USA; 4 Chittaranjan National Cancer Institute, Kolkata, 700026 India; 5 Division of Medical Oncology, Department of Internal Medicine, Ohio State University, Columbus, OH 43210 USA

**Keywords:** Collagen-induced arthritis, Rheumatoid arthritis, Angiogenesis, VEGF, Chebulinic acid, Treatment

## Abstract

**Background:**

Although vascular endothelial growth factor-A (VEGF)-induced angiogenesis has been reported to play an important role in the pathogenesis of rheumatoid arthritis (RA), serious side effects, mainly grade 2–3 hypertension, which is commonly observed with currently available anti-VEGF agents, can be detrimental for RA patients due to hypertension and associated cardiovascular complications seen in these patients. Thus, identification of anti-VEGF molecules that do not increase blood pressure could be useful for the treatment of RA. Chebulinic acid (CI), a water-soluble small-molecule tannin, can inhibit the actions of VEGF, and a report suggested that CI might not increase blood pressure due to its compensatory effects on the cardiovascular system. Therefore, the effects of CI on blood pressure in mice and the progression of the disease in a murine collagen-induced arthritis (CIA) model were investigated.

**Methods:**

CIA was induced in DBA/1J mice with type II collagen. The effects of CI in these animals were then evaluated by determination of clinical, histopathological, and immunohistochemical parameters. The effects of CI on VEGF-induced proangiogenic genes and signaling pathways were examined in vitro and in vivo.

**Results:**

Significant CD31 and VEGF expressions were detected in the synovial tissues of mice with CIA, similar to their expressions observed in human RA patients. However, treatment with CI significantly inhibited paw swelling, decreased the mean articular index and joint pathology scores in these animals through inhibition of VEGF-induced proangiogenic gene expressions and signaling pathways that regulate angiogenesis. Unlike currently used antiangiogenic agents, CI at a dose that inhibits VEGF actions did not increase blood pressure in mice.

**Conclusion:**

CI can act as a safe and potent anti-VEGF antiangiogenic agent for the treatment of types of inflammatory arthritis, such as RA.

**Supplementary information:**

The online version contains supplementary material available at 10.1186/s13075-020-02370-1.

## Introduction

Rheumatoid arthritis (RA) is a common inflammatory arthritis and significant cause of disability due to permanent joint destruction and deformity [1]. Several studies now indicate that angiogenesis is essential for the pathogenesis of RA [1–3]. Angiogenesis stimulates inflammatory cell infiltration and the development of invasive synovial pannus, which leads to the degradation of cartilage and subsequent joint damage [4]. Angiogenesis induced by VEGF, an angiogenic factor, has been shown to play a critical role in regulating this cascade in RA [4–9]. RA patients exhibit elevated VEGF levels in the serum, synovial fluid, and inflamed synovium [8, 9], which correlate with disease activity [9]. Therefore, targeting VEGF-mediated angiogenesis can be an effective therapeutic approach in RA patients [8, 9]. However, as reports from oncology clinics indicate that hypertension is a major side effect of currently used anti-VEGF agents and because RA patients commonly present with hypertension, it would therefore be prudent to identify an anti-VEGF antiangiogenic agent that does not increase blood pressure for the treatment of RA [10–13]. In this respect, chebulinic acid (CI), a naturally occurring tannin, may be a molecule of interest, because we recently demonstrated that CI could significantly inhibit the proangiogenic effects of VEGF on human umbilical vein endothelial cells (HUVECs) [14]. Another report indicated that CI has compensatory effects on the cardiovascular system, which in turn might prevent an increase in blood pressure [15]. Therefore, we investigated the efficacy of CI in a murine collagen-induced arthritis (CIA) model, a well-established preclinical model, to study the therapeutic efficacy of drugs for the treatment of types of inflammatory arthritis, such as RA [1, 16]. The effects of CI at a dose that inhibits VEGF on blood pressure in mice were determined.

## Materials and methods

### Cell culture and reagents

Mycoplasma-free and authenticated human synovial microvascular endothelial cells (HSMECs) procured from Cell Systems, WA, USA were cultured in complete medium supplemented with growth factor and 2% fetal bovine serum. For in vitro experiments, HSMECs were starved of serum and growth factor for 12 h before the effects of > 90% pure CI (Natural Remedies, Bangalore, India) were evaluated [14]. Recombinant human vascular endothelial growth factor-A (rhVEGF) was purchased from R&D Systems (MN, USA). All other chemicals were obtained from Sigma (MO, USA).

### CIA mouse model

Twelve 7- to 8-week-old male DBA/1J mice from Jackson Laboratories (USA) were used. The institutional IACUC committee approved all the procedures carried out. First, incomplete Freund’s adjuvant (IFA), which is a mixture of 15% Arlacel A and 85% mineral oil, was prepared (Sigma); then, heat-killed *Mycobacterium tuberculosis* (BD Bioscience, CA, USA) was added to IFA at a final concentration of 4 mg/ml to make complete Freund’s adjuvant (CFA). CFA and type ІІ collagen (Chondrex, WA, USA) were emulsified at a 1:1 ratio by a tissue homogenizer to make the final emulsion for injection. Fifty microliters of this emulsion was injected intradermally (i.d.) into the tail of each mouse at approximately 1.5 cm distal to the base of the tail [17]. The thickness of each affected hind paw was measured with microcalipers [18]. The paws of each mouse were clinically scored with the following scale of 0–4: 0 = normal, 1 = slight erythema and edema, 2 = increased edema with the loss of landmarks, 3 = marked edema, and 4 = marked edema with ankylosis of the joint. Finally, the articular index, which is the sum of the scores for all four paws of each mouse, was determined [19].

### Treatment

At 8 weeks after immunization, 12 mice with an articular index of approximately 3.5 were chosen and randomly assigned into two groups; the mice in one group received CI dissolved in sterile water at a dosage of 50 mg/kg/day orally by gavage for 14 consecutive days, while the mice in the control group received only sterile water. This dose of CI was selected because by liquid chromatography-tandem mass spectrometry (LC-MS/MS), we observed 1.7 μM CI to be the concentration of CI in the plasma of mice after their treatment with a single oral dose of 50 mg/kg of CI. Furthermore, our previous study also indicated that CI at this concentration (1.7 μM) could inhibit the proangiogenic actions of VEGF in vitro [14]. The articular index of each mouse was evaluated at the end of the treatment. The mice were then euthanized on the last day of our follow-up, i.e., on day 20, and all limbs with adjacent joints were harvested, fixed in 10% neutral buffered formalin, decalcified in 14% EDTA, and embedded in paraffin [19].

### Histological staining and scoring

Histopathological changes in joint tissue sections stained with hematoxylin and eosin were scored by two board-certified pathologists in a blinded manner. Cell infiltration was graded on a scale of 0–3 based on the number of inflammatory cells in the synovial tissue. Cartilage destruction was graded on a scale of 0–3, with scores indicating changes ranging from the appearance of dead chondrocytes to the complete loss of articular cartilage. Bone erosion was graded on a scale of 0–5, with scores indicating bone ranging from that with a normal appearance to that with a completely eroded cortical bone structure in the patella and femur condyle [20].

### Immunohistochemistry

The Institutional Review Board (IRB) committee at our institution approved the study of de-identified human synovial tissue sections of healthy human subjects and RA patients. Human knee and mouse ankle joint tissue sections were incubated with human or mouse primary antibodies against CD31 (10 μg/ml) (Cat# ab28364, Abcam, 1:100) or VEGF (0.5 μg/ml) (Cat# sc-152, Santa Cruz Biotechnology, 1:200) at 4 °C overnight and then with the corresponding secondary antibodies. Images were taken using an Axio Scope upright light microscope (Carl Zeiss, Oberkochen, Germany) and compared. Microvessel density (CD31 expression) or angiogenesis was quantitated by analyzing 10 random fields/section [21].

### Immunofluorescence

Paraffin-embedded tissue was baked and deparaffinized before antigen retrieval. After 1 h of blocking with normal donkey serum, tissues were incubated with two unconjugated primary antibodies, anti-CD31 (2 μg/ml) (Cat# sc-1506, Santa Cruz Biotechnology,1:100) and anti-phospho-vascular endothelial growth factor receptor-2 (p-VEGFR-2) (7.5 μg/ml) (Cat# ab5473, Abcam, 1:100) or anti-endothelial cell-specific molecule 1 (ESM 1) (10 μg/ml) (Cat# ab103590, Abcam, 1:100) and anti-Apelin (1.3 μg/ml) (Cat# 11497-1-AP, Life Technologies, 1:100) at 4 °C overnight. The corresponding fluorochrome-conjugated secondary antibodies were applied for 1 h at room temperature on the next day, and DAPI was used to counterstain the nuclei. Slides were mounted and visualized under a confocal scanning microscope (FBV-1000; Olympus Corporation, Center Valley, PA) [22].

### Western blot analysis

HSMEC lysates were collected and used for Western blot assays to determine the expression of phospho-extracellular signal-regulated kinases 1/2 (p-Erk1/2) (0.19 μg/ml) (Cat# 9101, Cell Signaling Technology, 1:1000), total Erk1/2 (0.019 μg/ml) (Cat# 9102, Cell Signaling Technology, 1:1000), phospho-Akt (p-Akt) (0.01 μg/ml) (Cat# 9271, Cell Signaling Technology, 1:1000), total Akt (0.034 μg/ml) (Cat# 9272, Cell Signaling Technology, 1:1000), phospho-p38 mitogen-activated protein kinase (p-p38 MAPK) (0.033 μg/ml) (Cat# 4511, Cell Signaling Technology, 1:1000), and total p38 MAPK (0.023 μg/ml) (Cat# 9212, Cell Signaling Technology, 1:1000). Finally, densitometry was used to quantitate the antibody-reactive bands [14, 21].

### Measurement of BP

Systolic and diastolic blood pressure (BP) in control (sterile water-treated) DBA/1J mice and DBA/1J mice treated with CI (50 mg/kg/day orally for 14 consecutive days by gavage) were measured using a computerized tail-cuff system (CODA system, Kent Scientific) [23].

### Hematology and blood biochemistry

Blood was collected by cardiac puncture from saline water-treated control mice and mice treated with CI (50 mg/kg/day orally for 14 consecutive days by gavage). Complete blood counts were undertaken in EDTA anticoagulated whole blood (FORCYTE Autosampler10, Oxford Science, Oxford, CT), and biochemical parameters were performed on serum samples (VetACE, Alfa Wasserman, West Caldwell, NJ) [23].

### Statistical analysis

Experimental data are expressed as the mean ± SEM. Student’s *t* test and ANOVA were used to analyze data from experiments involving independent groups. For experiments in which data were measured across the whole course of treatment, ANOVA with repeated measures was conducted [24]. Differences for which *p* < 0.05 were considered to be significant [14, 22]. Bonferroni correction was performed for multiple comparisons [25].

## Results

### Expression of VEGF and CD31 in the synovium of healthy human subjects and RA patients

**Fig. 1:**
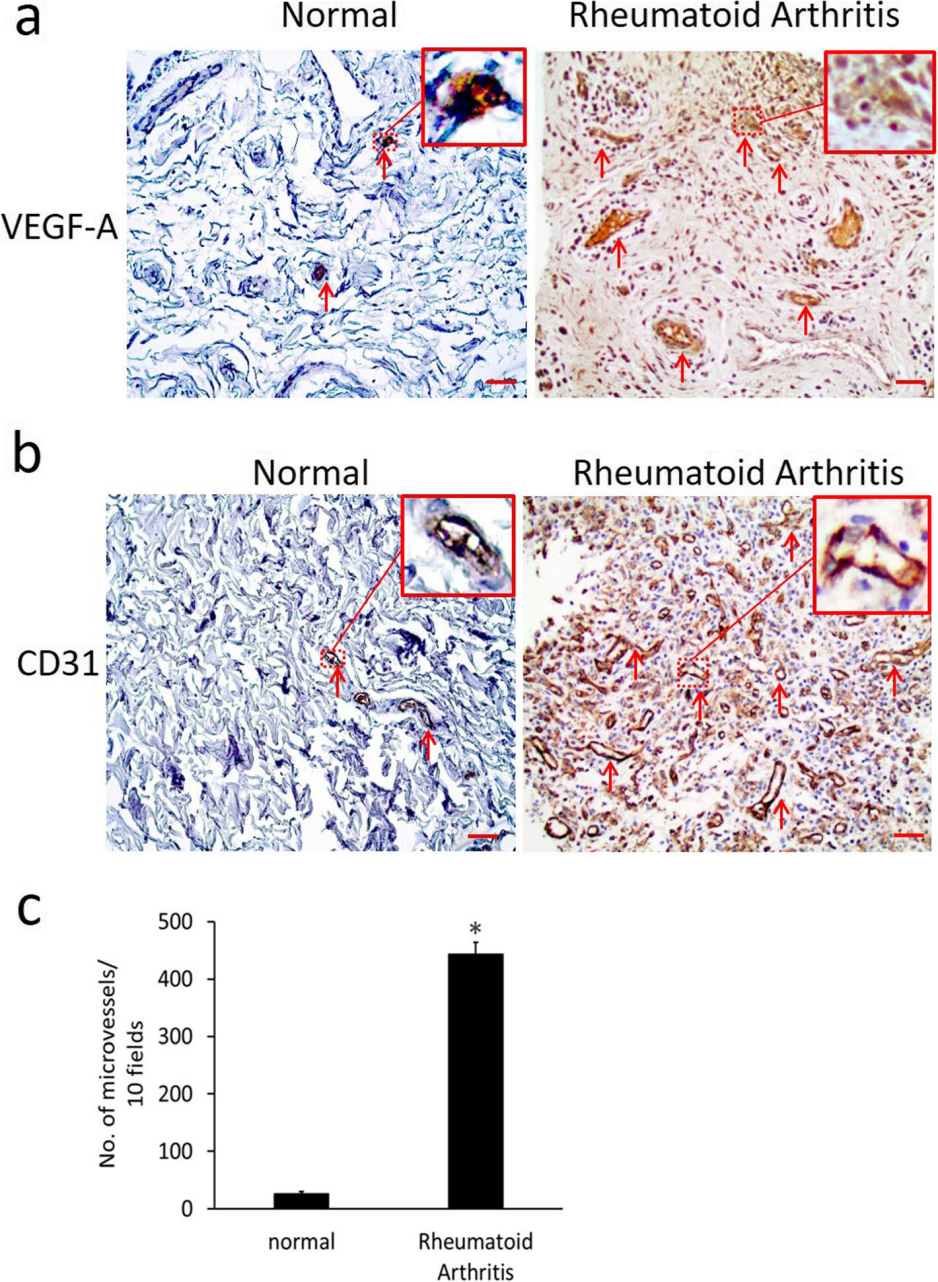
Immunohistochemistry staining shows **a** vascular endothelial growth factor-A (VEGF). **b** CD31 expressions in the synovial membranes collected from normal human and rheumatoid arthritis (RA) patients. **c** CD31 expressions are significantly increased in human RA synovial membranes (data are expressed as mean ± SEM, *P < 0.05 versus normal, Student’s *t* test.). Scale bar = 100 μm. The figure is a representative image from 6 normal and 6 RA patients with similar results

Since VEGF-induced angiogenesis has been reported to play an important role in the progression of RA, a type of inflammatory arthritis, we investigated VEGF and CD31 (a marker of angiogenesis) expression in human synovial membrane samples collected from de-identified age-matched (40–60 years) healthy human subjects (3 females and 3 males) who had undergone amputation due to injury and RA patients (3 females and 3 males) who underwent joint replacement surgery. The RA patients were treated with methotrexate. Our immunohistochemistry results demonstrated considerably more VEGF (Fig. 1a) and significantly increased CD31 (Fig. 1b and c; *p < 0.05) expression in the synovium of RA patients in comparison to normal controls, confirming VEGF-induced angiogenesis in the synovium of RA patients.

### Effect of CI on disease progression

**Fig. 2:**
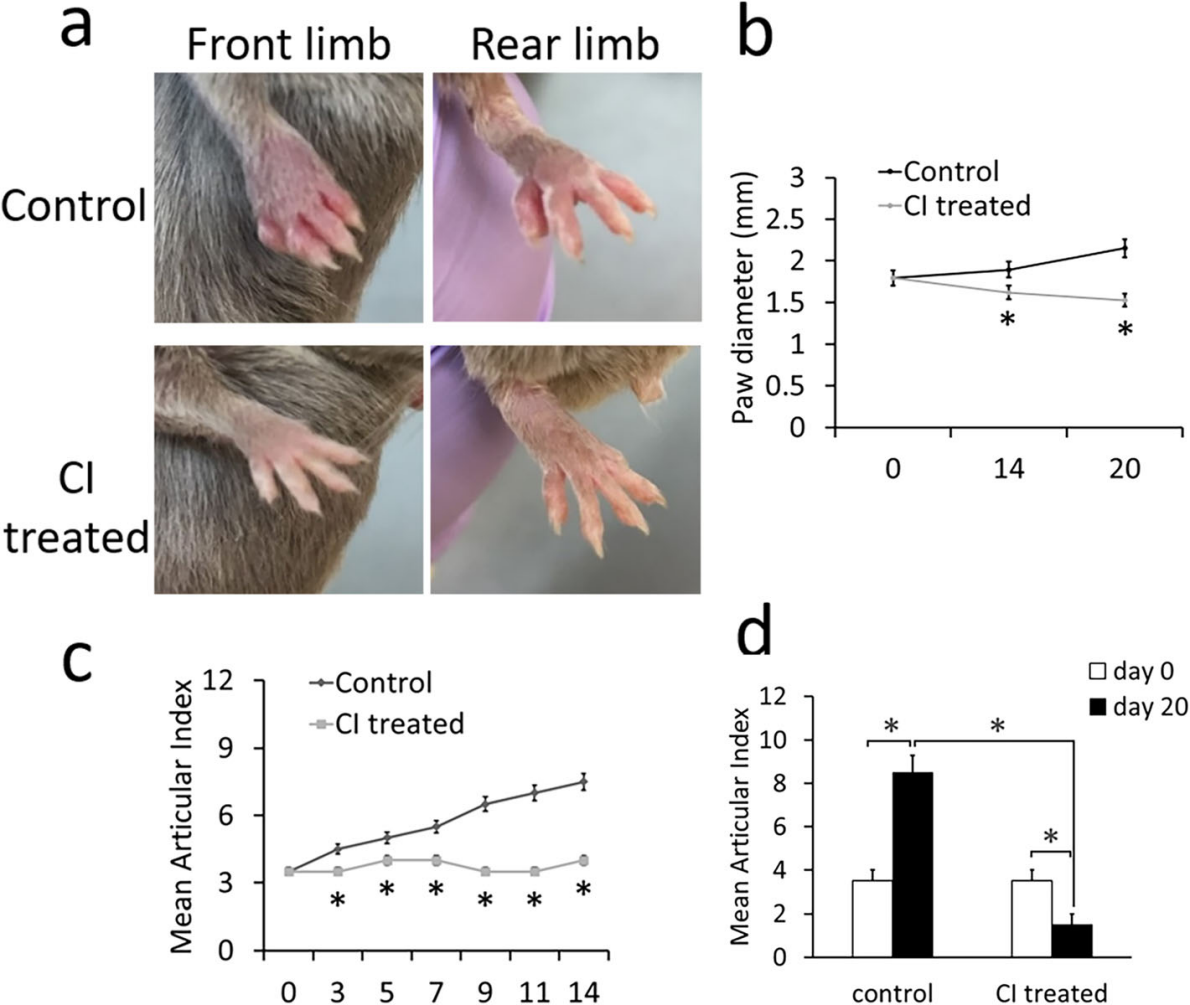
Effects of chebulinic acid (CI) treatment (50 mg/kg once daily orally for 14 days) on clinical disease progression in mice with collagen-induced arthritis (CIA). **a** Considerable reduction of joint erythema and swelling of both front and rear limbs. **b** Mean paw diameter in CI-treated mice compared to untreated control mice (data are expressed as mean ± SEM; *p < 0.05 versus control, ANOVA with repeated measures). **c** Mean articular index at the beginning and end of CI treatment, i.e., from days 0–14, comparing to untreated control (data are expressed as mean ± SEM; *p < 0.05 versus control, ANOVA with repeated measures). **d** Significantly reduced mean articular index in CI-treated mice compared to the untreated control group on day 20, i.e., on day six after completion of treatment (CI vs control at day 20, *p < 0.05). Mice untreated had increased mean articular index (day 20 vs 0, *p < 0.05), while mice treated with CI had reduced articular index (day 20 vs 0, *p < 0.05). Data are expressed as mean ± SEM, ANOVA with repeated measures. The figure is representative of 12 (6 = untreated control mice; 6 = CI-treated control mice) separate experiments undertaken in different animals with similar results

Compared with untreated control arthritic mice, mice orally treated with CI (50 mg/kg/day for 14 consecutive days) showed significantly inhibited CIA severity, as assessed by gross paw swelling (Fig. 2a), mean paw diameter (Fig. 2b, *p < 0.05), and mean articular index (Fig. 2c; *p < 0.05). Furthermore, on follow-up, the mean articular index in the CI-treated group on day 20, i.e., on day 6 after the completion of treatment, was significantly decreased compared to that in untreated controls (Fig. 2d; *p < 0.05). This dose and treatment schedule had no effects on the weight of animals.

**Fig. 3:**
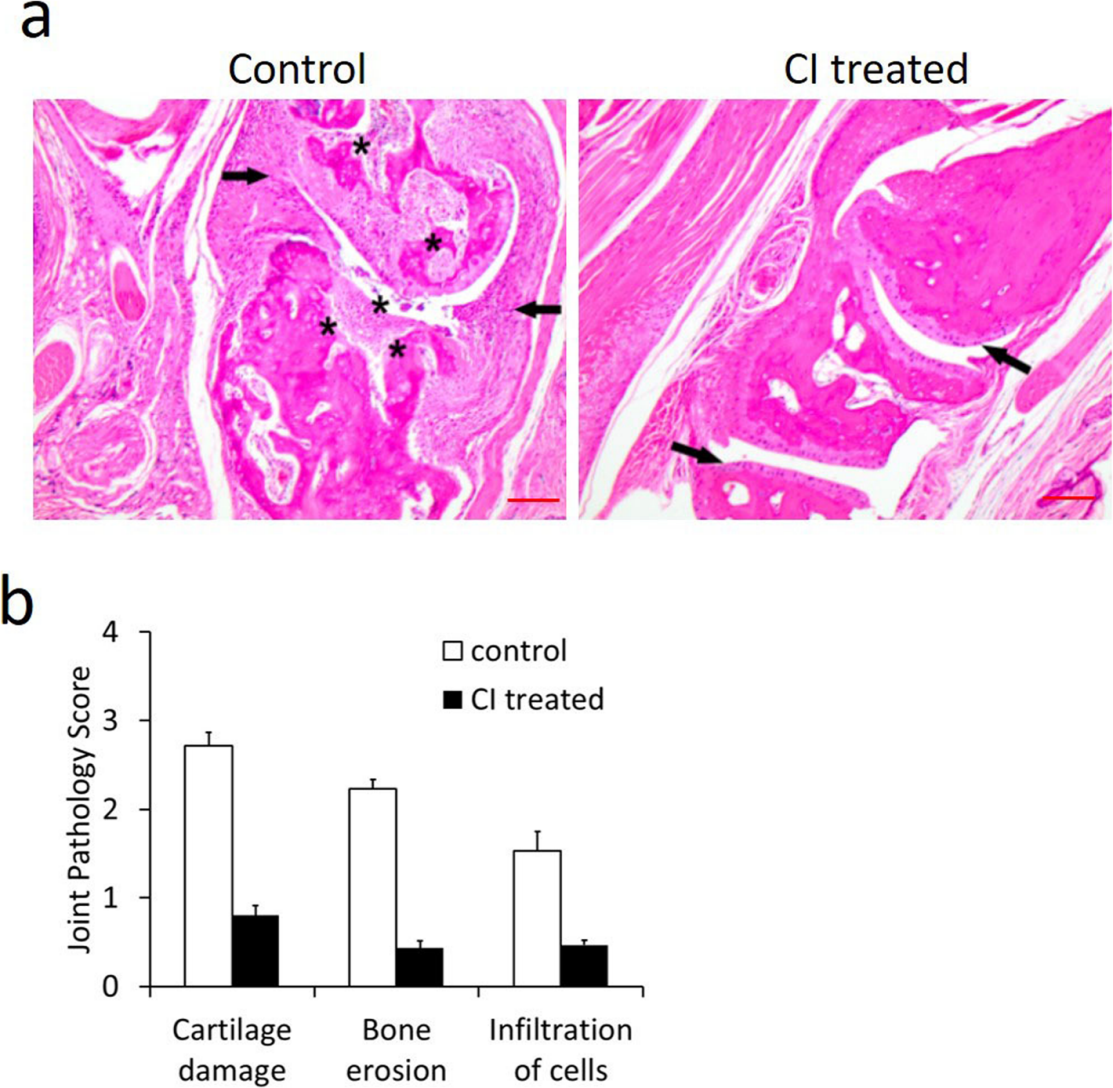
Hematoxylin and eosin (H&E) stained joint sections and semi-quantitative analysis of joint pathology scoring in mice with collagen-induced arthritis. **a**, **b** The arrows in the CI untreated control photomicrograph demonstrate high inflammatory milieu composed of polymorphous acute and chronic inflammation. The asterisks show extensive bone and cartilaginous destruction. On the contrary, the arrows in the CI (50 mg/kg once daily orally for 14 days)-treated joint tissues show preservation of the osteocartilaginous microanatomy with considerably less degenerative changes and inflammation. Scale bar = 100 μm. The figure is representative of 12 (6 = untreated control mice and 6 = CI-treated mice) separate experiments undertaken in different animals with similar results

Similarly, histopathological examination of hematoxylin- and eosin-stained sections of joint tissues and semiquantitative joint pathology scoring on day 20 showed chronic inflammation in the synovial membrane (infiltration of mononuclear cells) and bone and cartilaginous destruction in untreated control mice with CIA. In contrast, only residual erosive cartilaginous destruction with considerably less inflammatory infiltrate was observed in the mice treated with CI (Fig. 3a and b). These results, therefore, suggest that CI is a potent inhibitor of disease activity in mice with CIA.

### VEGF-induced angiogenesis in mice with CIA treated with CI

**Fig. 4:**
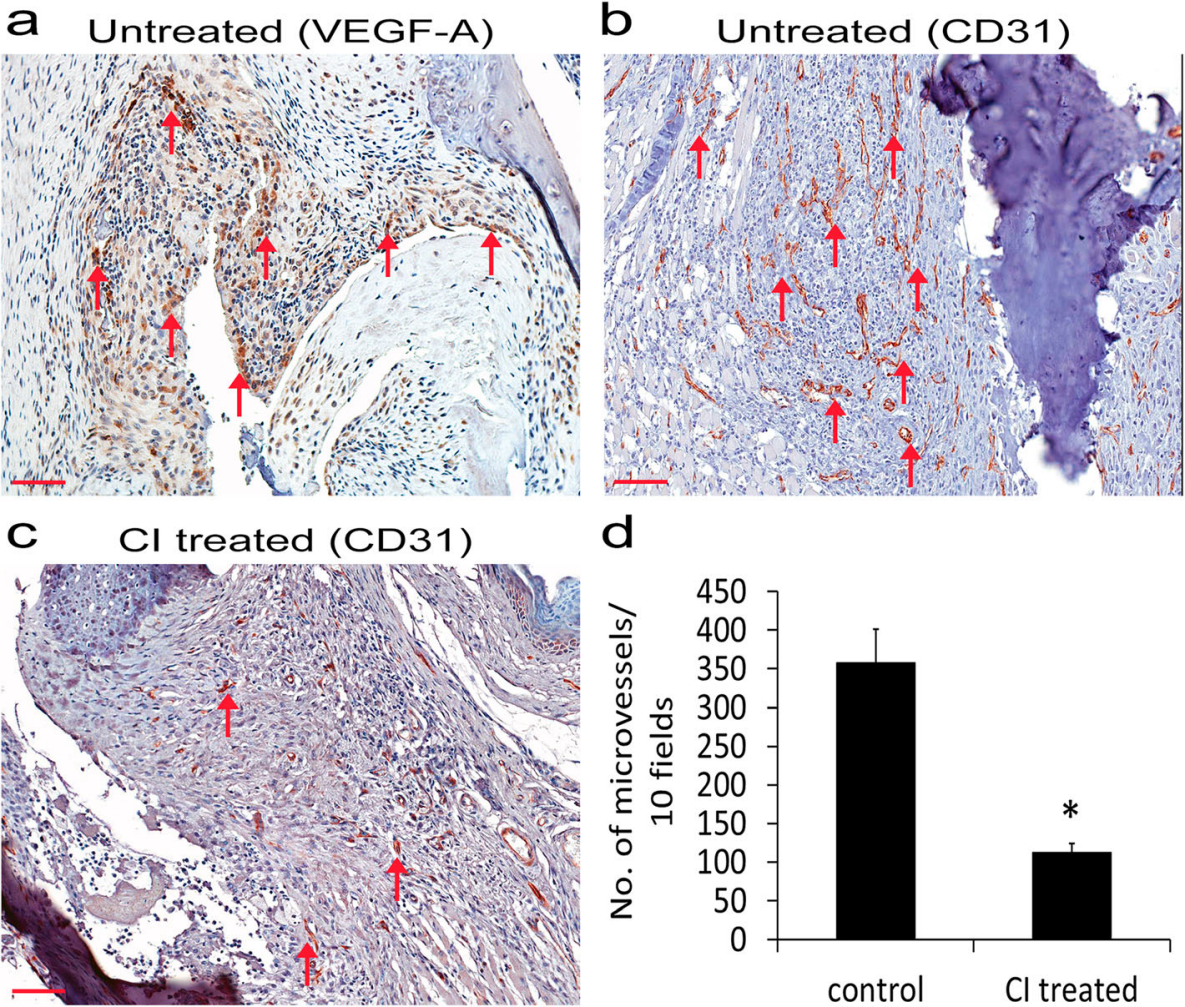
Immunohistochemistry staining of vascular endothelial growth factor A (VEGF) and CD31 in synovial membranes collected from collagen-induced arthritis (CIA) mice. **a** VEGF (red arrows) is expressed in the synovial membrane of mice with CIA. **b** CD31 staining (red arrows), i.e., the number of microvessels in untreated control CIA mice. **c** CD31 staining (red arrows), i.e., the number of microvessels in CI (50 mg/kg once daily orally for 14 days) treated CIA mice. **d** Significantly reduced CD31 staining, i.e., the number of microvessels in CI-treated mice in comparison to the CI untreated control group (data are expressed as mean ± SEM; *P < 0.05 versus control, Student’s *t* test). Scale bar = 100 μm. The figure is representative of 12 (6 = untreated control mice; 6 = CI-treated control mice) separate experiments undertaken in different animals with similar results

Our immunohistochemistry results indicated high VEGF (Fig. 4a) and CD31 (Fig. 4b) expression in the synovium of untreated animals; however, treatment with CI significantly inhibited CD31 expression (Fig. 4b–d; *p < 0.05), suggesting that CI can significantly inhibit angiogenesis in the synovial tissues of mice with CIA. However, treatment with CI had no effects on the VEGF expression (data not shown).

### Effects of CI on VEGF-dependent signaling and proangiogenic genes in synovial endothelial cells in vivo

**Fig. 5:**
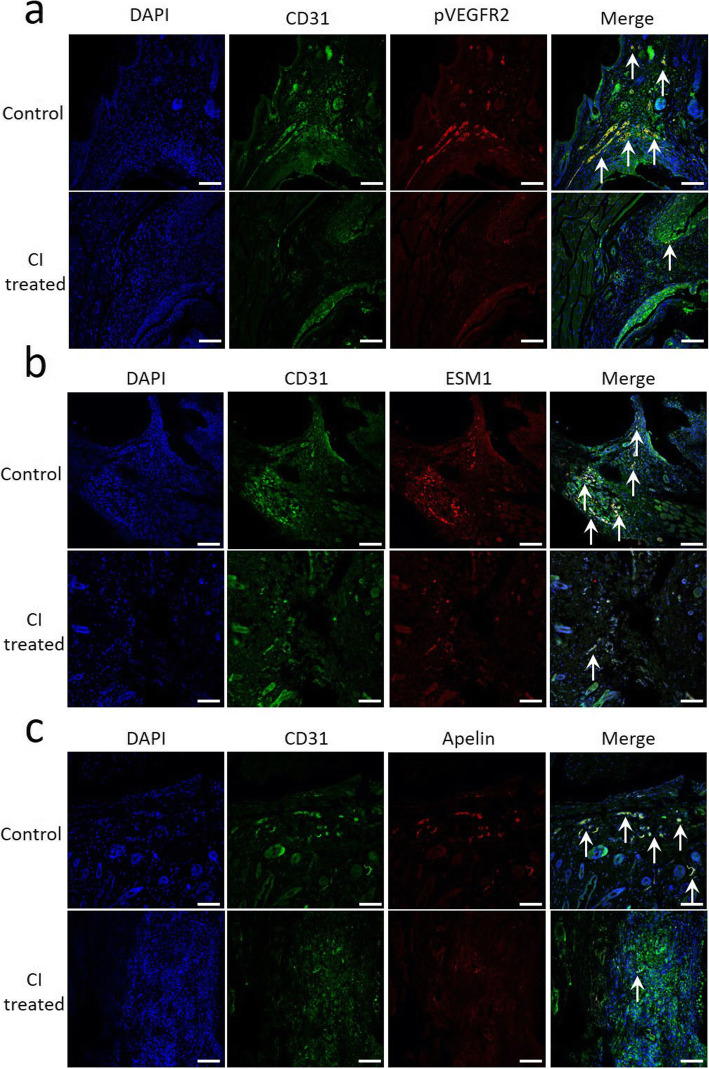
Co-localization of CD31 and VEGFR-2 signaling pathway markers in both chebulinic acid (CI) untreated and CI-treated collagen-induced arthritis (CIA) mouse joint tissues. **a** Immunofluorescence staining and confocal microscope were used to collect co-staining of CD31 (green), and pVEGFR2 (red) in both untreated and CI-treated CIA mouse joint tissues. **b** Immunofluorescence staining and confocal microscope were used to collect co-staining of CD31 (green), and ESM 1 (red) in both untreated and CI-treated CIA mouse joint tissues. **c** Immunofluorescence staining and confocal microscope were used to collect co-staining of CD31 (green) and Apelin (red) in both untreated and CI-treated CIA mouse joint tissues. Scale bar = 100 μm. The figure is representative of 12 (6 = untreated control mice; 6 = CI-treated control mice) separate experiments undertaken in different animals with similar results

To investigate whether the anti-arthritic actions of CI are specifically mediated through inhibition of VEGF-mediated angiogenesis in vivo, we first examined the effects of CI treatment on vascular endothelial growth factor receptor-2 (VEGFR-2) phosphorylation, as VEGF binds VEGFR-2 receptors in vascular endothelial cells to induce angiogenesis [22]. We then investigated the effects of CI treatment on expression of the VEGF-dependent vascular genes ESM 1 and Apelin, which are well-established surrogate markers of VEGF signaling, in vivo [26]. The results of our immunofluorescence colocalization experiment demonstrated significant inhibition of VEGFR-2 phosphorylation (Fig. 5a) and downregulation of ESM 1 (Fig. 5b) and Apelin (Fig. 5c) in the endothelial cells of CI-treated synovial tissues from CI-treated mice with CIA compared to untreated controls (See Supplemental Fig. 1 for isotype control). These results, therefore, confirm that CI acts specifically through inhibition of VEGF signaling in vivo.

### VEGF-mediated signaling pathways following CI treatment that regulate the angiogenic functions of endothelial cells

**Fig. 6:**
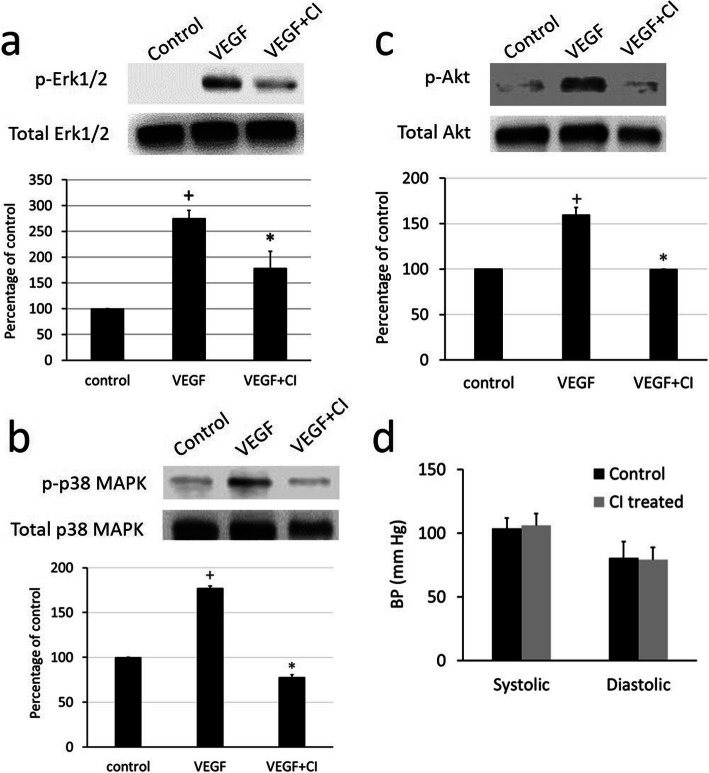
Effects of chebulinic acid (CI) treatment on vascular endothelial growth factor A (VEGF)-mediated downstream signaling pathways in human synovial microvascular endothelial cells. **a**–**c** Western-blot analyses show that in comparison to the untreated control, VEGF can induce significant Erk1/2 (~ 42 kD), p38 MAPK (~ 43 kD), and Akt (~ 60 kD) phosphorylation (*n* = 6, data are expressed as mean ± SEM; VEGF vs control, ^*+*^*P* < 0.05, one-way ANOVA). However, CI treatment significantly inhibits VEGF-mediated phosphorylation of Erk1/2, p38, and Akt (n = 6, Data are expressed as mean ± SEM. VEGF+CI vs VEGF, ^*^P < 0.05, one-way ANOVA). The blots were re-probed respectively with total Erk1/2 (~ 42 kD) or p38 MAPK (~ 40 kD) or Akt (~ 60 kD) antibodies. Figures are representative of six separate experiments under similar conditions. **d** CI treatment shows no significant changes in systolic and diastolic blood pressure in mice (*n* = 6) when compared to untreated controls (*n* = 6)

VEGF activates or phosphorylates its downstream signaling pathways, the Erk1/2, p38 MAPK, and Akt pathways, in endothelial cells (ECs) to induce the proliferation and migration of ECs and microvascular hyperpermeability, which are essential steps of angiogenesis [22, 27]. We investigated the effects of 1.7 μM CI on 10 ng of rhVEGFA-induced Erk1/2, p38 MAPK, and AKT phosphorylation in synovial microvascular ECs in vitro. Our results indicated that CI treatment significantly (*p < 0.05) inhibited VEGF-induced Erk1/2 (Fig. 6a), p38 MAPK (Fig. 6b), and AKT (Fig. 6c) phosphorylation in these cells compared with the untreated control cells. These results indicated that CI could inhibit the critical signaling pathways through which VEGF controls the angiogenic functions of ECs (See Supplemental Fig. 2 for full blots). It would be important to mention here that 2 μM CI had no direct effects on the viability of endothelial cells [14]. We had used a lower concentration of CI (1.7 μM) for these in vitro experiments.

### Effects of CI on BP

Since hypertension is the most commonly observed side effect of the anti-VEGF agents currently used in the clinic [10, 11], we investigated the effects of CI treatment on BP. We did not observe any significant difference in systolic or diastolic BP between CI-treated and untreated mice (Fig. 6d). These results indicate that CI at a dose that inhibits VEGF-induced angiogenesis does not increase blood pressure.

### Hematological effects of CI

As both neutropenia and thrombocytopenia have been reported with anti-VEGF drugs [28], we, therefore, determined the neutrophil and platelet counts in the CI-treated mice. Our results indicated that CI treatment does not cause neutropenia and thrombocytopenia (Table 1).

**Table 1:** Toxicity Studies

Parameters	Untreated normal mice (***n*** = 6)	Chebulinic acid-treated normal mice (***n*** = 6)
**Hematological parameters**		
Hemoglobin (g/dL) normal range, 11.0–15.1	13.0 + 1.02	13.0 + 0.8
Neutrophils (k/μL) normal range, 0.1–2.4	0.9 + 0.01	1.0 + 0.04
Platelet (k/μL) normal range, 592–2972	663.2 + 34.6	689.3 + 32.8
**Liver function tests**		
Alanine aminotransferase (ALT) [U/L] normal range, 16–50	24.3 + 1.8	24.7 + 2.0
Aspartate aminotransferase (AST) [U/L] normal range, 34–102	63.0 + 8.4	59.3 + 7.2
Total bilirubin (mg/dL) normal range, 0–0.3	0.23 + 0.01	0.21 + 0.01
**Renal function tests**		
Blood urea nitrogen (BUN) normal range, 14–32	20 + 2.0	19.5 + 1.5
Creatinine normal range, 0.1–0.6	0.23 + 0.03	0.2 + 0.01

### Effects of CI on liver function tests

Alanine aminotransferase (ALT) and aspartate aminotransferase (AST) elevations are reported in patients treated with angiogenic inhibitors [29]. However, our results indicated no significant differences in the ALT, AST, and bilirubin levels in vehicle and CI-treated animals (Table 1).

### Renal effects of CI

There is an association between VEGF inhibitors and renal damage [30]. In contrast to the presently used anti-VEGF agents, there were no significant changes in the serum blood urea nitrogen (BUN) and creatinine levels in CI and vehicle-treated mice (Table 1).

## Discussion

Although VEGF-induced angiogenesis plays a pivotal role in the pathogenesis of RA (Fig. 1), the anti-VEGF agents that are currently used to treat cancer patients have serious side effects, especially grade 2–3 hypertension, which may limit the use of these agents in RA patients [10–13]. Several studies now indicate that RA is associated with hypertension; this common cardiovascular risk factor is more prevalent in RA patients than in the general population, leading to increased morbidity and mortality from cardiovascular disease in these patients [12, 13]. Therefore, the need to identify anti-VEGF molecules devoid of this side effect is essential.

CI is a water-soluble small-molecule tannin [14]. Interestingly, our results indicated that CI could significantly inhibit paw swelling and decrease the mean articular index and joint pathology score in mice with CIA compared with untreated control mice, suggesting that CI could retard disease activity in these animals (Figs. 2 and 3). Furthermore, hypertension is the most common side effect of currently available anti-VEGF agents; recent reports indicate that the incidence of hypertension after the use of these agents varies between 20 and 87% [10, 11]. Interestingly, our results showed that unlike currently used anti-VEGF agents, CI at a dose that inhibits VEGF-induced angiogenesis in vivo did not increase BP (Fig. 6d). This action of CI might be due to its ability to decrease cardiac output as a consequence of reduced left ventricular contraction [15]. CI also had no toxic effects on the CBC, liver function tests, BUN, and creatinine levels (Table 1) and weight of the animals. However, it will be necessary to evaluate the chronic toxic effects of CI in the future.

Moreover, CD31 expression and angiogenesis were also significantly inhibited in CI-treated animals with CIA compared to untreated controls (Fig. 4b–d). Importantly, our results confirmed for the first time that CI specifically inhibits the proangiogenic actions of VEGF in vivo (Fig. 5) [26]. Since it is now well established that the biological properties of ECs in different organs vary considerably and because our previous studies were undertaken in human umbilical vein endothelial cells [14, 31, 32], in the present study, we investigated the effects of CI on VEGF-mediated downstream signaling pathways in synovial microvascular ECs. Our results demonstrated for the first time that CI could significantly inhibit VEGF-induced Erk 1/2, p38 MAPK, and AKT phosphorylation in ECs (Fig. 6a–c), the members of critical downstream signaling pathways through which VEGF controls the proliferation and migration of ECs and microvascular permeability, essential steps through which VEGF mediates angiogenesis [22, 27].

Although CI has an effect on the VEGF pathway, further research is required to fully characterize the molecular mechanisms by which CI slows the progression of the CIA phenotype. Previous observations showed that two other polyphenols with different chemical structures, chebulagic acid and chebulanin, which are both extracted from the plant *Terminalia chebula* Retzius, also exhibit antiarthritic effects [19, 33]. Chebulagic acid suppresses the onset and progression of CIA through immune suppression by inducing TGFβ and CD4+ and CD25+ T cells. Chebulanin, on the other hand, mediates its antiarthritic effect in mice with CIA by suppressing the expression of inflammatory mediators and preventing cartilage destruction and bone erosion. Notably, these three polyphenols are derivatives of 2,4-O-chebuloyl-d-glucose [34]. Therefore, it would be of interest to eventually perform a comprehensive study on the molecular mechanisms through which these three polyphenols suppress the symptoms and progression of arthritis in a CIA mouse model. It is possible that an optimal therapeutic effect will be attainable by combining these polyphenols with other drugs already in use.

## Conclusions

In summary, this study indicates for the first time that CI, a water-soluble, orally bioavailable small molecule [14], could significantly improve disease activity in mice with CIA by inhibiting VEGF-induced angiogenesis through molecular mechanisms not reported before. Moreover, since hypertension and associated cardiovascular complications are common in RA patients and because our results demonstrate that CI does not increase blood pressure [12, 13], clinical trials may be undertaken in the future to evaluate the efficacy of CI in RA patients, either alone or in combination with other agents currently used for the treatment of RA.

## Supplementary information

**Additional file 1.**

**Additional file 2.**

## Data Availability

The authors are committed to sharing their data, publishing the data, and making available the resources described in this publication to the scientific community.

## Abbreviations

VEGF: Vascular endothelial growth factor-A
RA: Rheumatoid arthritis
CI: Chebulinic acid
HUVEC: Human umbilical vein endothelial cells
CIA: Collagen-induced arthritis
HSMECs: Human synovial microvascular endothelial cells
rhVEGF: Recombinant human vascular endothelial growth factor-A
IACUC: Institutional Animal Care and Use Committee
IFA: Incomplete Freund’s adjuvant
CFA: Complete Freund’s adjuvant
I. D.: Intradermally
IRB: Institutional Review Board
p-VEGFR2: Phospho-vascular endothelial growth factor receptor-2
LC-MS/MS: Liquid chromatography-tandem mass spectrometry
ESM 1: Endothelial cell-specific molecule 1 (ESM 1)
p-Akt: Phospho-Akt
p-p38 MAPK: Phospho-p38 mitogen-activated protein kinase
BP: Blood pressure
EDTA: Ethylenediaminetetraacetate
