# Supplementary material for: Chebulinic acid is a safe and effective antiangiogenic agent in collagen-induced arthritis in mice
Source: Arthritis Res Ther. 2020 Nov 23;22:273. doi: 10.1186/s13075-020-02370-1 (PMC7682078; doi:10.1186/s13075-020-02370-1)
Supplement: Supplementary file 1 — Additional file 1. [file 13075_2020_2370_MOESM1_ESM.zip › supplementary Figure 1 legend.pdf]

Supplementary Fig. 1 Goat IgG (Cat# 31245, Life Technologies, 1:100) and rabbit IgG (Cat# 026102, Life Technologies, 1:100) were used as isotype control, DAPI for nuclear staining.
