# Supplementary figures and images for: Chebulinic acid is a safe and effective antiangiogenic agent in collagen-induced arthritis in mice
Source: Arthritis Res Ther. 2020 Nov 23;22:273. doi: 10.1186/s13075-020-02370-1 (PMC7682078; doi:10.1186/s13075-020-02370-1)

Supplemental Fig. 1

Isotype  
IgG with  
DAPI

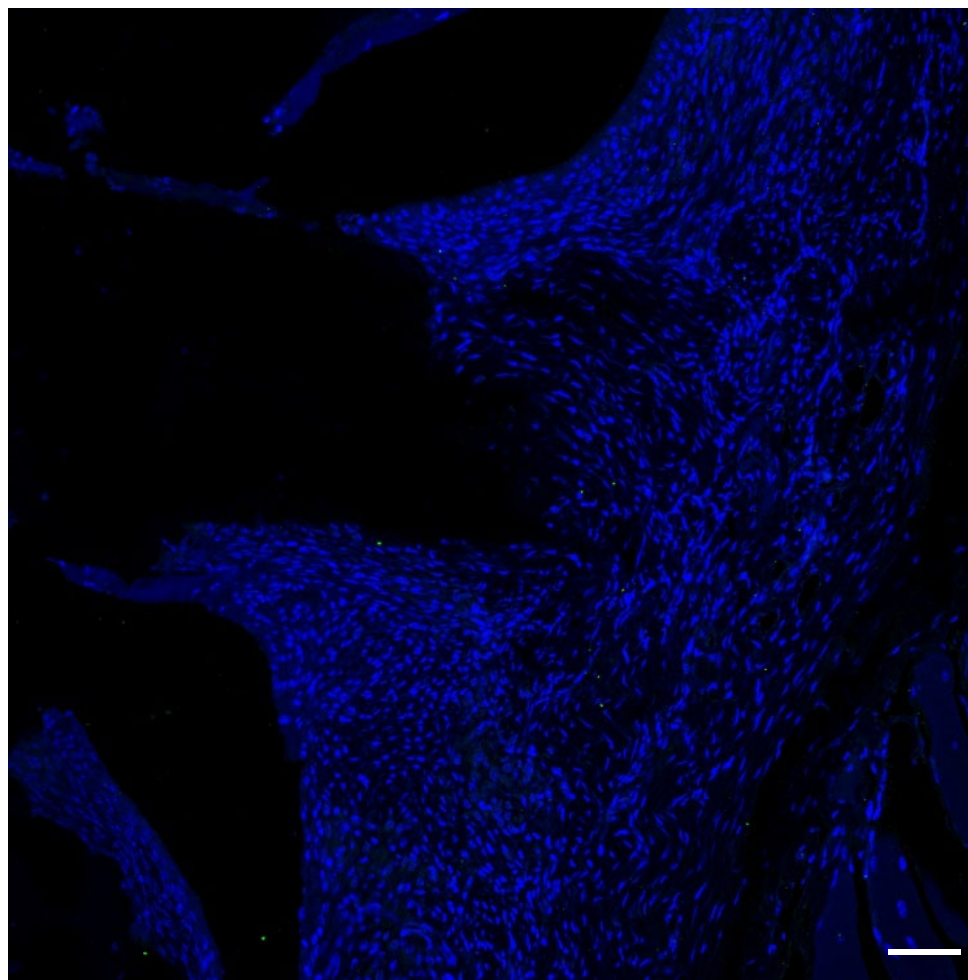

Supplement: Supplementary file 1 — Additional file 1. [file 13075_2020_2370_MOESM1_ESM.zip › supplementary Figure 1.pdf]
