# Supplementary material for: Chebulinic acid is a safe and effective antiangiogenic agent in collagen-induced arthritis in mice
Source: Arthritis Res Ther. 2020 Nov 23;22:273. doi: 10.1186/s13075-020-02370-1 (PMC7682078; doi:10.1186/s13075-020-02370-1)

Figure 6 a

P-Erk1/2:

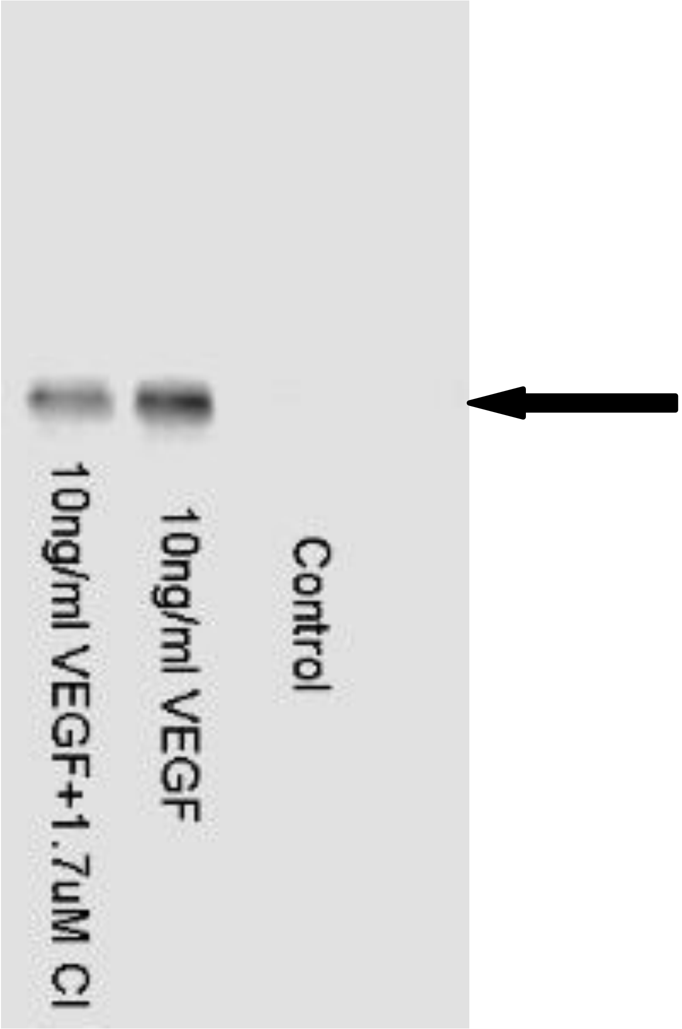

Figure 6 a

Total Erk1/2:

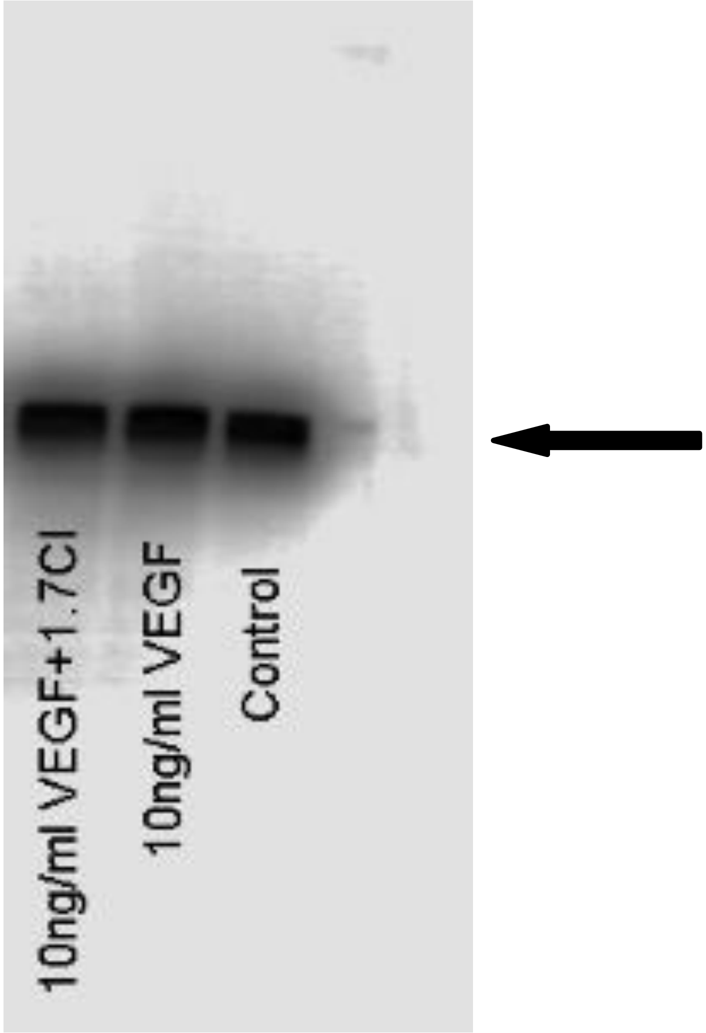

Figure 6 b

p-p38 MAPK:

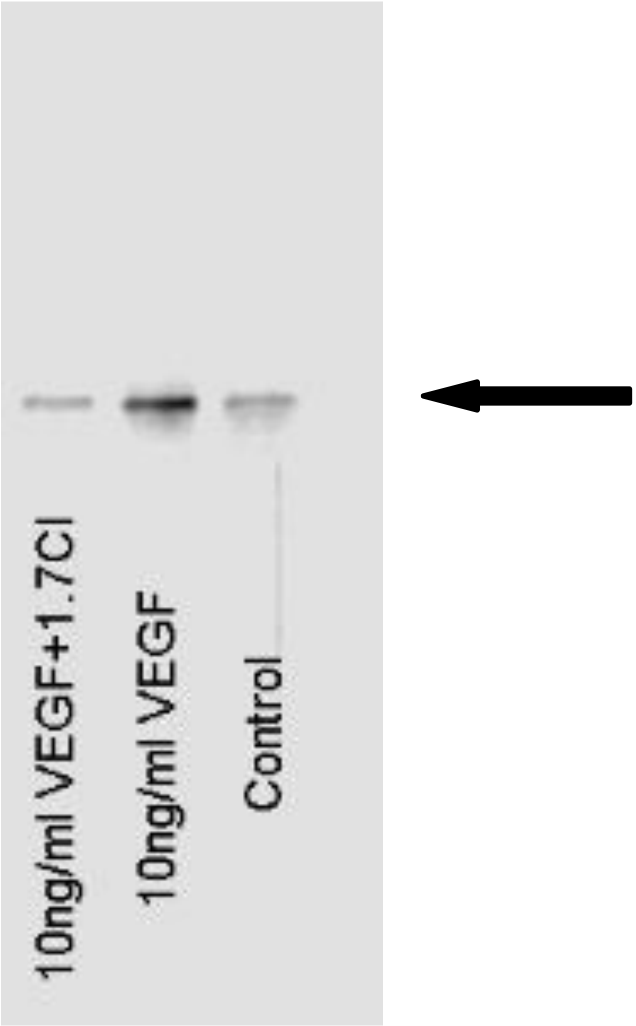

Figure 6 b

Total p38 MAPK:

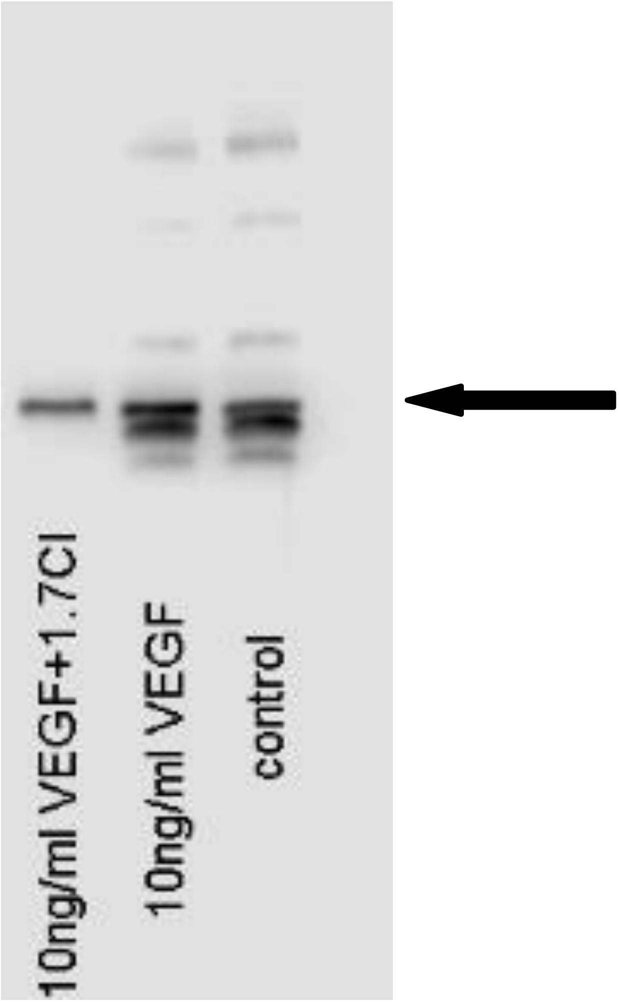

Figure 6 c

p-Akt:

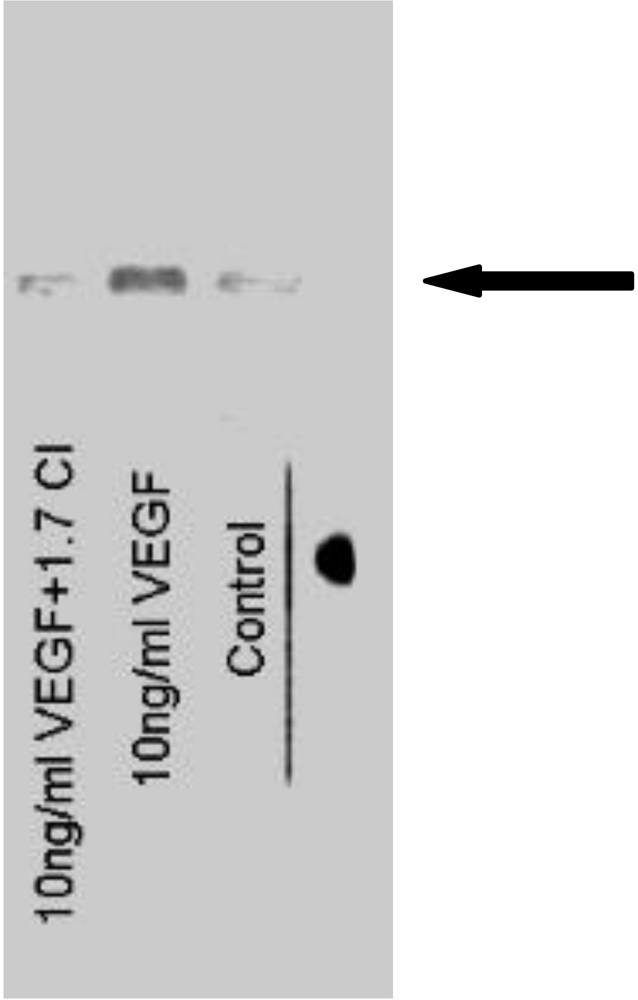

Figure 6 c

Total-Akt:

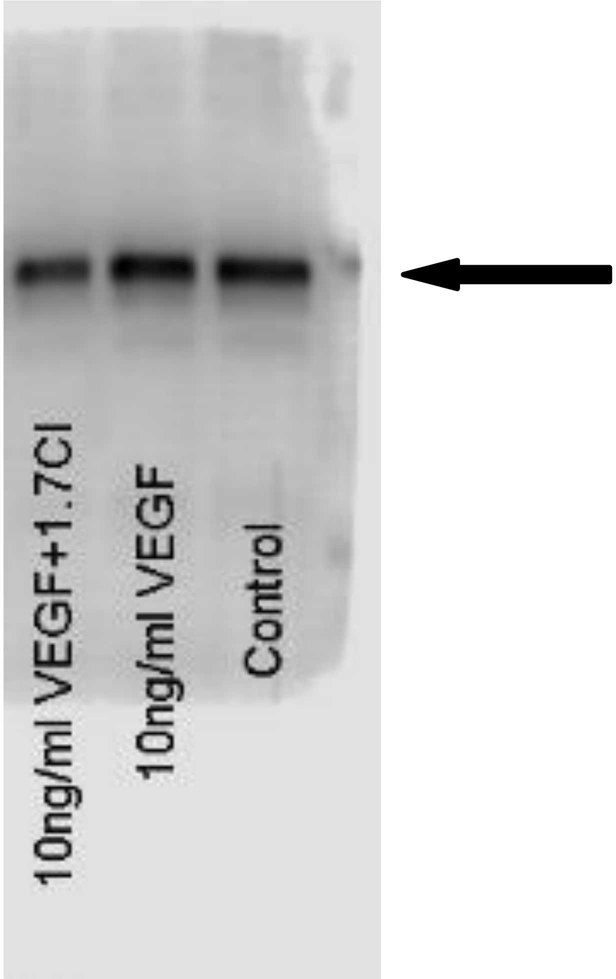

Supplement: Supplementary file 2 — Additional file 2. [file 13075_2020_2370_MOESM2_ESM.pdf]
